# Effects of Diesel Exhaust Particles on Mouse Gastric Stem Cells

**DOI:** 10.3390/life10080149

**Published:** 2020-08-12

**Authors:** Heba Al-Sadik, Subi Sugathan, Prashanth Saseedharan, Shahrazad Sulaiman, Sumaya Beegam, Abderrahim Nemmar, Samir Attoub, Sherif M. Karam

**Affiliations:** 1 Department of Anatomy, College of Medicine & Health Sciences, United Arab Emirates University, P.O. Box 17666, Al-Ain, UAE; 201370005@uaeu.ac.ae (H.A.-S); subisugathan@uaeu.ac.ae (S.S.); prash1987@hotmail.com (P.S.); 2 Department of Pharmacology and Therapeutics, College of Medicine & Health Sciences, United Arab Emirates University, P.O. Box 17666, Al-Ain, UAE; sharazadjeffy@uaeu.ac.ae (S.S.); samir.attoub@uaeu.ac.ae (S.A.); 3 Department of Physiology, College of Medicine & Health Sciences, United Arab Emirates University, P.O. Box 17666, Al-Ain, UAE; sumayab@uaeu.ac.ae (S.B.); anemmar@uaeu.ac.ae (A.N.); 4 Zayed Center for Health Sciences, United Arab Emirates University, P.O. Box 17666, Al Ain, UAE

**Keywords:** stem cells, diesel exhaust particles, cell viability, cell migration, oxidative stress

## Abstract

Stem cells have attracted many scientists because of their unique properties and therapeutic applications. However, very little is known on the environmental toxins that could affect their biological features. This study focuses on the consequences of the exposure of a cell line representative of the mouse gastric stem/progenitor (mGS) cells to diesel exhaust particles (DEPs). These immortal cells were cultured using routine protocols. The DEPs were added to the culture media at 1, 10, and 100 µg/mL for 1 to 72 h. The cells were assayed for their viability, migration, oxidative stress, and the expression of genes specific for cell proliferation, pluripotency, and death. DEPs induced a reduction in the metabolic activity of mGS cells, only at a high concentration of 100 µg/mL. However, no significant effects were detected on cell migration, oxidative stress markers (glutathione and thiobarbituric acid reactive substances), and cell death related proteins/genes. Interestingly, these findings were associated with down-regulation of Notch 2 and 3 and Bmi-1 proteins and activation of STAT3 involved in the regulation of the fate of stem cells. In conclusion, this study demonstrates that mGS cells have some resistance to oxidative stress and apoptosis when exposed to DEPs at the expense of their stemness.

## 1. Introduction

The emissions of diesel exhaust particles (DEPs) from engines represent a major predisposing factor for diseases affecting the cardiovascular, respiratory, urinary, and nervous systems [1,2,3,4,5]. In the digestive system, DEPs induce several adverse effects in rat liver cells: activation of aryl hydrocarbon receptors, phosphorylation of checkpoint kinases and p53 during the cell cycle, and formation of DNA adducts and breaks [6]. Therefore, DEPs contribute to increasing rates of both morbidity and mortality around the world [7].

The DEPs are composed of a core of elemental carbon with adsorbed organic compounds such as polyaromatic and nitro-polyaromatic hydrocarbons, heavy metals, gaseous materials, and some trace elements. DEPs also include micro- and nano-particles with a huge surface area that can cross the air–blood barrier in the lungs [8].

Surprisingly there is no information about the cellular and molecular effects of DEPs on the stomach, and especially, it is very likely that some of these environmental particles are not only inhaled but can get also trapped in the saliva and swallowed to reach the stomach. However, an epidemiological study has demonstrated a significant correlation between occupational exposure to diesel exhaust and stomach cancer [9]. The epithelial lining of the stomach includes dividing stem cells capable of maintaining themselves and differentiating into cell lineages producing mucus, acid, pepsinogen, and hormones. Therefore, these stem cells are responsible for the homeostasis of the gastric epithelium throughout the life of the organism [10,11,12].

Little is known about the gastric epithelial progenitor/stem cells in health and disease. Some studies have demonstrated that alterations in their proliferation and differentiation programs occur during development of gastric dysplasia and neoplasia and, therefore, might contribute to the origin of gastric cancer [13,14,15,16]. Moreover, some evidences indicate that *Helicobacter pylori* binding to these cells and decreased gap junctions are major factors involved in the pathogenesis of peptic ulcers, gastritis, and even adenocarcinoma [17,18]. Thus, further studies on these progenitor/stem cells are necessary for better understanding of the pathogenesis of several stomach diseases, including gastric cancer, one of the leading causes of cancer deaths worldwide [19]. The high frequency and mortality rates of gastric cancer in many regions around the world are warning signals to improve our basic information on the stem/progenitor cells and factors that might affect their behavior.

With the advancement of knowledge about stem cells and their applications in tissue engineering and regenerative medicine, it is not known how environmental toxins and/or particles might affect their biological features in health and disease. Even though DEPs can reach the stomach and get into direct contact with gastric epithelial lining including stem cells, no information is available about the effects of these particles on the gastrointestinal stem cells. Therefore, the overall aim of this study was to investigate the effects of environmental DEPs on gastric epithelial stem cells using immortalized, cloned adult mouse gastric stem (mGS) cells [14] with a special focus on i) cell proliferation and migration, ii) oxidative stress, and iii) the expression levels of some genes or proteins regulating the stemness of gastric stem cells.

## 2. Materials and Methods 

### 2.1. Preparation of DEPs

The DEPs (SRM 2975) were obtained from the National Institute of Standards and Technology (Gaithersburg, MD, USA). DEPs (1000 µg) were suspended in 1 mL sterile saline (0.9% NaCl) containing 0.01% Tween 80. To minimize aggregation of particles, the suspensions of DEPs were sonicated for 15 min and vortexed immediately before use. For control, saline containing 0.01% Tween 80 was used. These particles were previously analyzed by a transmission electron microscope and shown to have a substantial amount of ultrafine (nano) sized particle aggregates and larger particle aggregates [20,21]. 

### 2.2. Cell Culture

Frozen aliquots of the mGS cells of passages 20–25 were cultured in T75 flasks using RPMI 1640 media containing 10% fetal bovine serum (Hyclone, Cramlington, UK) and antibiotics (penicillin 50 U/mL; streptomycin 50 µg/mL) at 37 °C incubator adjusted to 5% CO^2^ [14]. These immortal stem/progenitor cells form spheres if grown in non-adhering conditions. The mGS cells require neither low oxygen nor antioxidants for their culture [22,23]. The culture media was changed every other day. When semi-confluent, the cells were passaged twice to stabilize their morphology and growth rate before being used in this study. The human lung cancer cells A549 were also maintained in RPMI 1640 supplemented with penicillin/streptomycin and 10% fetal bovine serum.

### 2.3. Cell Viability Assay

The mGS cells or A549 cells were plated in cell culture flask, then trypsinized and counted by a handheld automated cell counter (Merck-Millipore, Billerica, MA, USA). The cells were seeded in 96-well plates at 5000 cells/100 µL media per well. After 24 h of incubation the cells were treated with different concentrations of DEPs (0, 1, 10, 100 µg/mL). For control, cells were incubated with media containing only the vehicle. After 1, 6, 24, and 48 h, mGS cells with different concentrations of DEPs were incubated with 100 μL of CellTiter-Glo^®^ 2.0 reagent for 10 min at room temperature. The A549 cells were incubated the different Des concentration only for only one time point, 24 h. The amount of ATP released from the cells was quantified by using GloMax-Luminometer (Promega, Madison, WI, USA). The released ATP was taken to reflect the number of viable cells in each well [24]. The data were graphically presented as mean ± SEM using GraphPad Prism software (La Jolla, CA, USA).

### 2.4. Glutathione Measurement 

The mGS cells were seeded in 12-well plates at 100,000 cells/well/mL. After 24 h, fresh media containing different concentrations of DEPs (1, 10, 100 µg/mL) were added. For control, cells were cultured in media containing only the vehicle. After 24 h, 1 mL media (supernatant) from each well were deproteinized with 5% sulfosalicylic acid, centrifuged to remove the precipitated protein, then assayed for glutathione using dithionitrobenzoic acid (DTNB) method [25]. The assay was carried out in a 96 well plate. In the first 2 wells, 10 µl of the 5% 5-Sulfosalicylic acid was added as a blank. Then, 10 µl glutathione standard solutions were added in duplicate into separate wells. The samples were added also in duplicate into separate wells. Then 150 µl of the assay buffer containing potassium phosphate, EDTA, glutathione reductase, NADPH, DTNB were added to each well and mixed. After 5 min incubation at room temperature, 50 µl of the diluted NADPH solution were added. The presence of glutathione in the samples causes reduction of DTNB to a yellow product, 5-thio-2-nitrobenzoic acid (TNB), which was measured spectrophotometrically using a plate reader at 412 nm. The reaction rate was proportional to the concentration of glutathione. A standard curve of reduced glutathione was used to determine the amount of glutathione in the samples. The data were presented as mean ± SEM. 

### 2.5. Thiobarbituric Acid Reactive Substances (TBARS) Measurement 

From the culture media of mGS cells incubated with DEPs, 100 µL were transferred into 5 mL tubes for lipid peroxidation assay [26]. Equal volumes of standard solutions were added in separate tubes. Then, 100 µL of sodium dodecyl sulfate (SDS) solution were added to the samples and mixed well. The color reagent (4 mL) containing thiobarbituric acid, acetic acid, and sodium hydroxide was added forcefully in each tube. The sample-containing tubes were heated in a water bath at 95 °C. After one hour, the samples were placed on ice for 10 min to stop the reaction and then centrifuged for 10 min at 3000 rpm. Then, 150 µL of each sample were loaded in duplicate to 96-well plate to measure the absorbance by spectrophotometer at 490 nm. The data were presented as mean ± SEM. 

### 2.6. NO Measurement 

The nitrite/nitrate assay kit (Sigma, St Louis, MO, USA) was used for measurement of NO [27]. Briefly, all samples and standards were run in duplicate using a 96-well plate. Equal volumes (100 µl) of standard solutions (NaNO2 in H2O) and samples of the culture media of mGS cells incubated for 24 h with DEPs at different concentrations were transferred into separate wells. Then, 100 µL of the Griess reagent were added and all samples were incubated for 5–10 min at room temperature for the color reaction to develop. The absorbance was measured using BioTek reader EL×800 at 540 nm. The readings were proportional to the NO metabolite present in the samples. 

### 2.7. Cell Migration or Scratch Wound Healing Assay

The mGS cells were seeded in 6-well plates at 1.5 × 10^6^ cells/well. After 24 h of incubation, when the cells formed a confluent monolayer, a scratch wound was made along the middle of each well with a sterile pipette tip. Then, the media was removed, and the cells were washed with phosphate buffered saline (PBS). Media containing DEPs (1, 10 µg/mL) prepared as described above were added. For control, the vehicle was only added without any DEPs. After 1, 6, and 24 h, the cells with the scratch wound were photographed using inverted Olympus microscope and DP70 digital camera. For each well, the width of the wound was measured at three different places for each time point. The difference between the measurements at 0 h and the measurements at 1, 6, and 24 h were taken as the migration distance and expressed as mean ± SEM. The percentage of wound healing was also calculated and blotted against time.

### 2.8. Western Blot Analysis 

The mGS cells were seeded at the density of 2 × 10^6^ cells. After 24 h, cells were incubated with different concentrations of DEPs (1, 10, 100 µg/mL). After 24 h, the culture media were collected and the cells were lysed with RIPA buffer (Thermo Scientific, Rockford, IL, USA) and then centrifuged at 14,000 rpm for 20 min. For measurement of protein concentration, the bicinchoninic acid assay (Pierce, Rockford, IL, USA) and NanoDrop (ThermoFisher, Waltham, MA, USA) at 562 nm were used. 

Proteins were separated in SDS gel and transferred onto nitrocellulose membrane (Schleicher & Schuell BioScience, Dassel, Germany). The membranes were incubated with the blocking buffer (1×TBS in fat-free milk) for 1 h, then with primary antibodies diluted at 1:500 in TBS-Tween, then with horseradish peroxidase-conjugated donkey anti-rabbit IgG (Jackson ImmunoResearch, West Grove, PA, USA) diluted at 1:1000 in blocking buffer. The primary antibodies used were rabbit polyclonal anti-Notch 2, Notch 3, total Oct4, Bmi-1, Bcl2, cleaved caspase 3, pSTAT3 antibodies, and mouse monoclonal anti-Caspase 3, Notch 4, cleaved PARP, and STAT3 antibodies (Cell Signaling Technology Inc., Danvers, MA, USA). For A549 cells, protein samples were processed for SDS gel, Western blotting, and membranes were probed with antibodies specific for rabbit polyclonal anti-cleaved caspase 3, pSTAT3 antibodies and mouse monoclonal anti-Caspase 3, and STAT3 antibodies. The membranes were washed and probed with mouse monoclonal anti-β-actin antibody (Santa Cruz Biotechnology) as loading control. Immunoreactive proteins in the blots were detected using SuperSignal West Pico chemiluminescent substrate on CL-Xposure film (Thermo Scientific, Barrington, IL, USA).

### 2.9. Quantitative Real-time Polymerase Chain Reaction (qRT-PCR) 

The mGS cells were seeded in 12-well plates at 30,000 cells per well. After 24 h of incubation, the cells were incubated with fresh media containing different concentrations of DEPs (1, 10, 100 µg/mL). After 6, 24, and 48 h, cells were washed with PBS buffer and then processed for RNA extraction using RNeasy kit (Qiagen, Hilden, Germany). The RNA lysis buffer was added into each well. The lysates were collected into microcentrifuge tubes. Equal volume of 70% ethanol was added to each well. The mixture was transferred into RNeasy spin column and centrifuged at 10,000 rpm for 15 sec. The flow-through and collection tube were discarded. Spin column membrane was washed, and total RNA was eluted. The RNA was quantified using a NanoDrop spectrophotometer and stored at −80 °C.

The cDNA was synthesized using the GoScript reverse transcription kit (Promega, Madison, WI, USA) and a Veriti 96-well Thermal Cycler (Applied Biosystems, Foster City, CA, USA). So, 1 µg of RNA was added to the master mix containing random primers in PCR tubes. The tubes were heated at 70 °C for 5 min and then immediately chilled on ice. Then, 10 µL of the reverse transcription reaction mix was added to each tube. The reaction was carried out for annealing at 25 °C for 5 min and extension at 42 °C for 1 h followed by the inactivation of the reverse transcriptase enzyme at 70 °C for 15 min. Samples of the synthesized cDNA were stored at −20 °C.

For real-time PCR, the TaqMan method and the QuantStudio 7 Flex real-time PCR system (Applied Biosystems, Waltham, CA, USA) were used. Primers specific for BCL2, GSTP1, and KI67 genes were obtained from ThermoFisher. Reaction mixtures (25 μL) containing 2 μL of cDNA template, 1.5 μL each of primer and probe mix, and TaqMan Universal PCR master mix (Applied Biosystems) were processed as follows: denaturation at 95 °C for 10 min and 40 cycles at 95 °C for 10 s, 60 °C for 20 s. PCR products were monitored by measuring the fluorescence produced by the result of TaqMan probe hydrolysis after every cycle. The expressions of different genes were analyzed using the comparative ΔΔCT method and the fold differences were calculated using 2^−ΔΔCT^. The expression levels of the genes examined were determined in duplicate and normalized using the hypoxanthine phosphoribosyltransferase 1 gene.

### 2.10. Statistical Analysis 

The data from different assays were analyzed by using the one-way ANOVA with Dunnett’s multiple comparison test model. A P value of less than 0.05 was taken to indicate a significant difference. Graphical representation of the data (mean ± SEM) was performed using GraphPad Prism software (Version 8, GraphPad Software, San Diego, CA, USA) or Microsoft Excel 365 (Version 2019, Microsoft Corporation, Redmond, WA, USA).

## 3. Results

**Figure 1:**
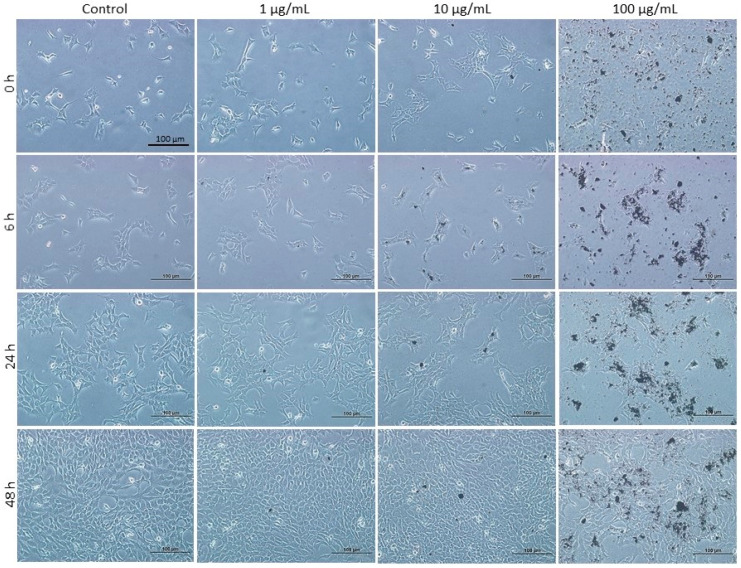
Light micrographs taken at the same magnification and showing mouse gastric stem (mGS) cells before (0 h) and after 6, 24, and 48 h of incubation without (0 µg/mL) and with different concentrations of diesel exhaust particles (DEPs) (1, 10, and 100 µg/mL) as shown with 20x objective lens. Note that control cells with 0 µg DEPs do not show apparent difference when compared with cells incubated with 1 or 10 µg/mL of DEPs after 6, 24, and 48 h. There are some round spaces between the attached cells when incubated with 100 µg of DEPs for 48 h. Some DEPs tend to form dark clumps at high concentrations. Magnification bar = 100 µm.

Incubation of mGS cells with different concentrations of DEPs for 6–48 h revealed minor morphological changes only at high concentrations. At 1 µg/mL DEPs, no apparent morphological changes were observed when compared with control (Figure 1). However, at 10 and 100 µg/mL, the density of attached cells was reduced, and some spaces appeared between the cells especially after 48 h of incubation (Figure 1).

### 3.1. Effect of DEPs on the Viability of mGS Cells

**Figure 2:**
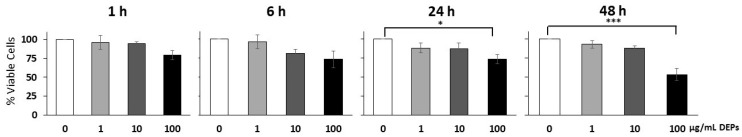
Cell viability assay for mGS cells after 1, 6, 24, and 48 h of incubation with DEPs at different concentrations (0, 1, 10, and 100 µg/mL). Cellular viability is significantly reduced only after 24 and 48 h of incubation with 100 µg DEPs. * *p* < 0.05, *** *p* < 0.001.

After 1 and 6 h of incubating mGS cells with different concentrations of 1, 10, and 100 µg/mL of DEPs, the cell viability assay showed a slight reduction in the percentage of viable cells. Statistical analysis showed that this decrease was not significant after 1 and 6 h of incubation (Figure 2). However, the percentages of viable mGS cells after 24 and 48 h of incubation with a high concentration (100 µg/mL) of DEPs showed a significant decrease (*p* < 0.05, 0.001, respectively) when compared with control cells (Figure 2). Nevertheless, at the lower concentrations of DEPs (1 and 10 µg/mL), there were slight reductions in the percentages of viable cells which were not statistically significant (Figure 2).

### 3.2. Effect of DEPs on the Expression of Apoptosis-related Proteins

**Figure 3:**
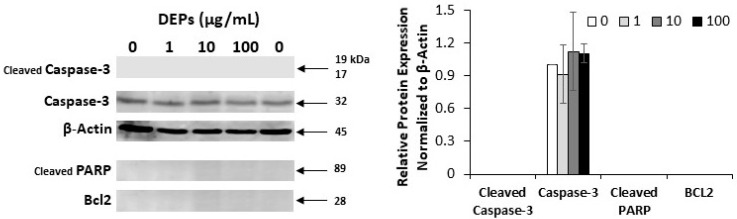
Western blots of mGS cells incubated with DEPs at 0, 1, 10, and 100 µg/mL for 24 h. Antibodies specific for apoptosis-related proteins: caspase-3, Bcl2, and the cleaved forms of caspase-3 and PARP were used. While the levels of caspase-3 did not show any significant change with DEPs incubations, Bcl2 and the cleaved forms of caspase-3 and PARP were not detected. Densitometric analysis of the protein bands is presented in the graph.

To verify whether the reduction in the number of mGS cells when incubated with DEPs is due to apoptosis, DEPs-treated mGS cells were processed for protein extraction and Western blot analysis using antibodies specific for pro-caspase-3 and cleaved caspase-3, cleaved PARP, and Bcl2. While there were no changes detected in the levels of caspase-3 (Figure 3), Bcl2 and the cleaved forms of PARP and caspase 3 were not detected in mGS cells (Figure 3). 

### 3.3. Effect of DEPs on Oxidative Stress Markers

**Figure 4:**
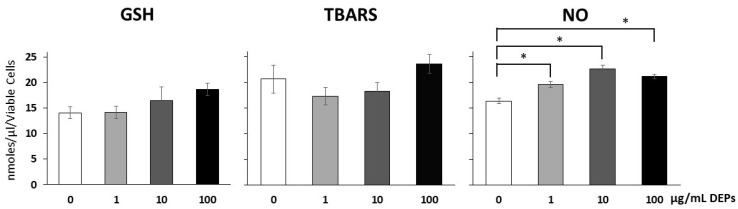
Average levels of glutathione (GSH), TBARS and NO released in the culture media of mGS cells incubated with different concentrations of DEPs: 0 (control), 1, 10, and 100 µg/mL for 24 h. No significant changes were detected in GSH and TBARS at any of the concentrations tested. The levels of NO released from DEPs-treated mGS cells were increased (* *p* < 0.05).

To test for oxidative stress, the levels of three different markers were averaged from three different experiments and each was run in triplicate. Measurements of glutathione released in the media of cultured mGS cells exposed to DEPs (1, 10, and 100 µg/mL) for 24 h revealed that the average values from three different experiments were 14.1, 16.5, and 18.7 nmol/µL, respectively. These values were not significantly different from each other or from the control value which averaged 14.1 nmol/µL (Figure 4). The levels of TBARS released in the media of mGS cells incubated with DEPs showed some changes when compared to control values (Figure 4). The averaged values of three different experiments were 20.7 nmol/µL in control and 17.4, 18.3, and 23.6 nmol/µL, respectively in cells exposed to DEPs at 1, 10, and 100 µg/mL (Figure 4). None of these changes in the levels of TBARS were significantly different from the control. As for NO, data of three different experiments were averaged and the level of released NO in culture media of control mGS cells was 16.3 nmol/µL. However, the average values for cells exposed to DEPs at 1, 10, and 100 µg/mL were 19.6, 22.6, and 21.2 nmol/µL, respectively. Statistical analyses indicated that this moderate increase was significant (Figure 4).

### 3.4. Gene Expression Analysis Using qRT-PCR

**Figure 5:**
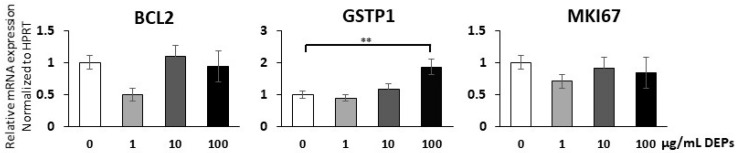
Quantitative RT-PCR showing relative changes of mRNA expression of BCL2, GSTP1, and MKI67 genes normalized to HPRT gene in mGS cells incubated with 1, 10, and 100 µg/mL of DEPs for 24 h when compared with the control (0 µg/mL DEPs). ** There is only a significant increase in GSTP1 expression at 100 µg/mL of DEPs.

To substantiate the above findings regarding apoptosis and oxidative stress and also to test for cell proliferation, RNA samples were extracted from mGS cells cultured in the presence of different concentrations of DEPs for 24 h. The RNA was subjected to cDNA synthesis and qRT-PCR analysis using primers specific for BCL2, GSTP1, and MKI67 genes. The gene expression levels were averaged from three different experiments and each was run in duplicate. The expression of the housekeeping gene HPRT showed consistent levels in mGS cells and, therefore, was used for normalization. The results showed no significant changes in the expression levels of BCL2 and MKI67 when compared to control (Figure 5). Incubation of mGS cells with 1 or 10 µg/mL of DEPs also did not show any significant change in the expression levels of GSTP1 gene (Figure 5). However, incubation with 100 µg/mL DEPs significantly up-regulated the level of GSTP1 expression (Figure 5). 

### 3.5. Effect of DEPs on the Migration of mGS Cells

**Figure 6:**
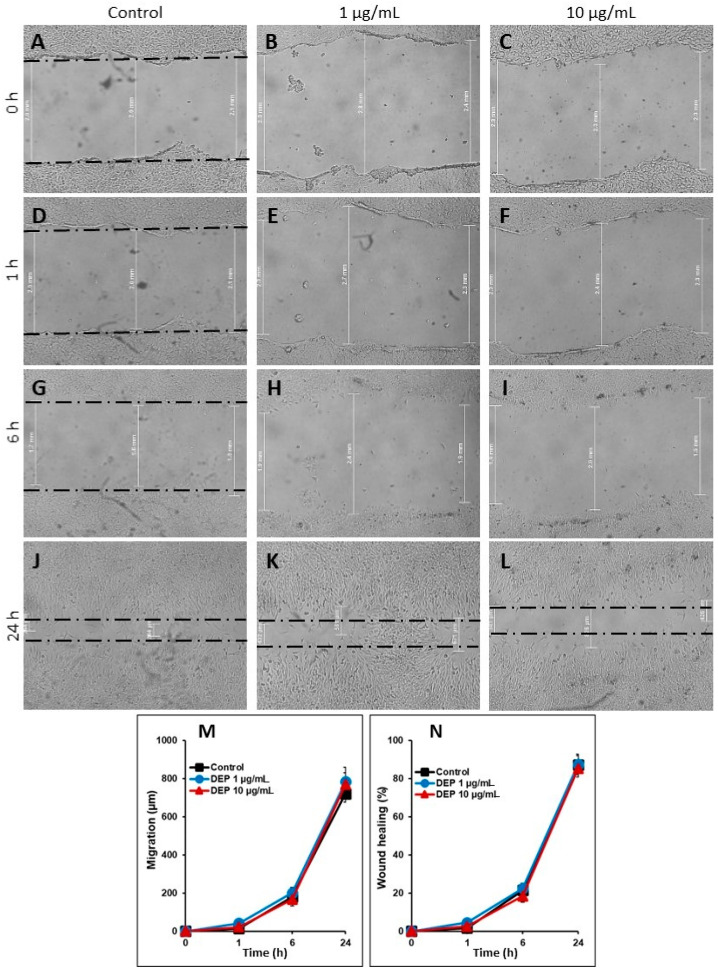
(**A**–**L**): Light micrographs showing the effect of DEPs (0, 1, and 10 µg/mL) on the migration of mGS cells after 0 (**A**–**C**), 1 (**D**–**F**), 6 (**G**–**I**), and 24 (**J**–**L**) h incubation. The widths of the wounds at each time point show little or no differences when compared with control cells (0 µg/mL). (**M**,**N**): Graphs showing the effect of DEPs on the migration and wound healing of mGS cells. The migration data from four different experiments (means ± SEM) are presented in graphs showing migration distance in micrometer (**M**) and percent of wound healing (**N**). In both graphs, the data show correlation between the values of control and cells incubated with DEPs at 1 and 10 µg/mL for 1, 6, and 24 h.

To investigate the effect of DEPs on cell migration, monolayers of mGS cells were scratched and DEPs at 1 and 10 µg/mL were added for 1, 6, and 24 h. Measurements of the widths of the wounds at each time point were recorded (Figure 6A–L). These cell migration data were expressed in two different ways (Figure 6M,N). First, the migration distance in µm of the mGS cells was blotted against time following 1, 6, and 24 h incubation with 0, 1, and 10 µg/mL DEPs (Figure 6M). In control wells, the migration distance gradually increased to reach about 750 µm at 24 h (Figure 6M). When mGS cells were incubated with either concentration of DEPs, we did not detect any significant effect on the speed of their migration at any of the time points. Second, the percentage of wound healing for each condition was calculated and blotted against time. Again, control cells showed a gradual increase in the percentage of wound healing reaching about 80% after 24 h (Figure 6N). Statistical analysis demonstrated no significant differences in the percent healing between control cells and those incubated with DEPs. Therefore, at the concentrations and time points examined, DEPs do not seem to affect the migration of mGS cells at least in this in vitro assay.

### 3.6. Effects of DEPs on the Expression of Stem Cell-related Proteins in mGS Cells

**Figure 7:**
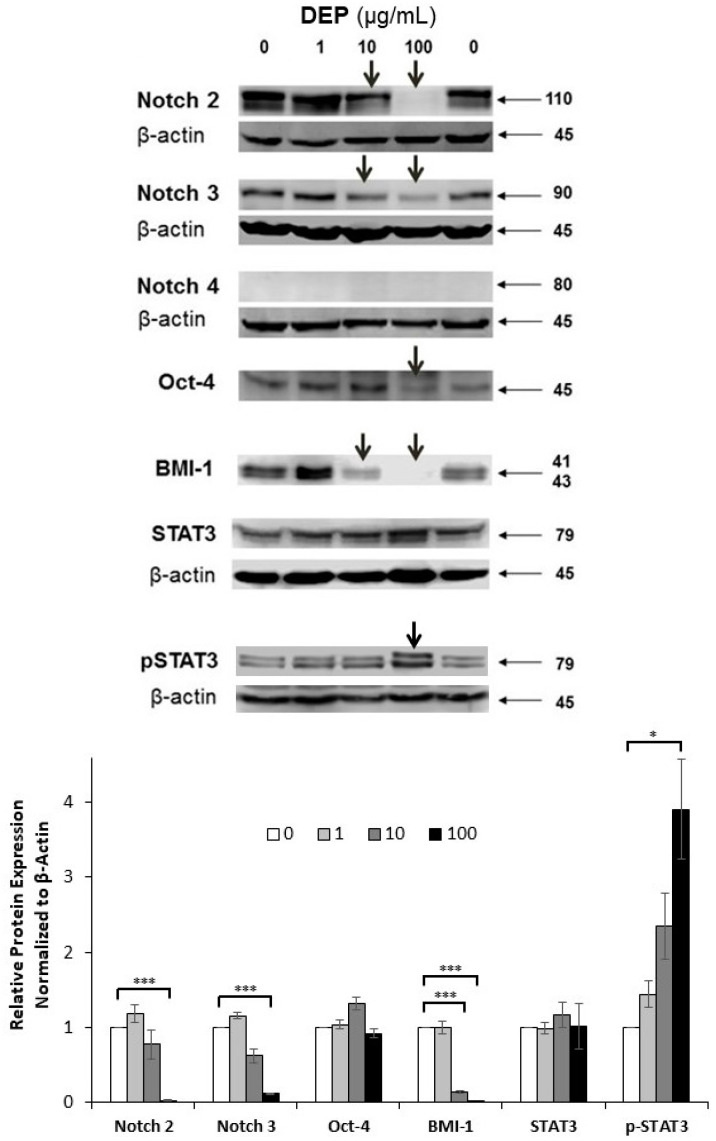
Protein expression analysis of stem cell-related genes following incubation of mGS cells with DEPs at 0, 1, 10, and 100 µg/mL for 24 h. Proteins were extracted and 30 µg from each sample were processed for SDS-PAGE and western blot analysis using antibodies specific for Notch 2, 3, and 4, Oct4, BMI-1, STAT3 and phosphorylated STAT3. Beta actin was used as the loading control. Note the down-regulations of Notch 2 and 3, Oct-4 and BMI-1, and up-regulation in the phosphorylated form of STAT3. Densitometric analysis of the protein bands is presented in the graph. * *p* < 0.05, *** *p* < 0.001.

To test whether the phenotype of mGS cells is affected following incubation with DEPs at 1, 10, and 100 µg/mL for 24 h, equal amounts of total cellular proteins (30 µg) were processed for western blot analysis using different antibodies previously detected in gastric stem cells, such as Notch 3, Oct-4 and BMI-1 [12,28,29]. When membranes were probed with Notch antibodies, it was interesting to reveal a change in their expression patterns (Figure 7). This was apparent with Notch 2 and 3 where a gradual decrease in the band density with increased concentration of DEPs was demonstrated. Notch 4 was not detected in mGS cells. When probing was carried out using antibodies specific for Oct4 and BMI-1, there was an apparent down-regulation at the concentration of 100 µg/mL DEPs (Figure 7). This might suggest that mGS cells were differentiating.

Because of their role in stem cell fate and differentiation, and because of the down-regulation of the growth rate of mGS cells following DEPs incubation, protein expression of STAT3 was also tested to determine whether it is altered with DEPs’ incubation. Probing by using two different antibodies specific for STAT3 and its phosphorylated form, showed an increased expression of STAT3 and its tyrosine phosphorylation following treatment with DEPs in a dose dependent manner (Figure 7).

### 3.7. Effects of DEPs on A549 Lung Cells

**Figure 8:**
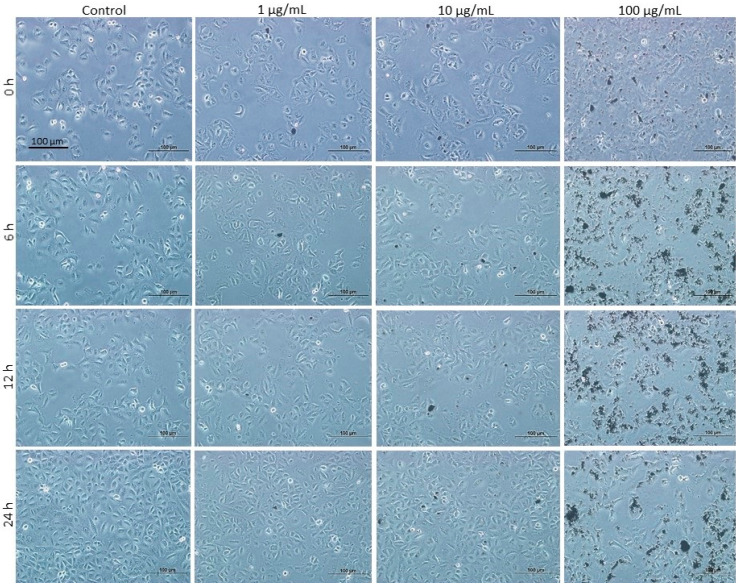
Light micrographs taken at the same magnification showing A549 cells following incubation with different concentrations of DEPs, 0 (control), 1, 10, and 100 µg/mL for 0, 6, 12, and 24 h. Note that control cells with 0 µg DEPs do not show apparent difference when compared with cells incubated with 1 or 10 µg/mL of DEPs after 6, 12, and 24 h. There are some round spaces between the attached cells when incubated with 100 µg of DEPs for 24 h. Some DEPs tend to form dark clumps at high concentrations. Magnification bar = 100 µm.

To test whether the cellular and molecular effects observed for DEPs are specific for mGS cells, we used another type of cells, lung epithelial A549 cell line that have some characteristics of stem/progenitor cells [30]. We first examined their morphology following incubation with 1, 10, and 100 µg/mL DEPs. As shown in Figure 8, at 1 and 10 µg/mL DEPs concentration, the cells appeared healthy with no apparent difference from the control and reached semi-confluence after 24 h of incubation. However, with the 100 µg/mL DEPs, the cells did not reach semi-confluency after 1 day and wide spaces were apparent in between the cells. 

**Figure 9:**
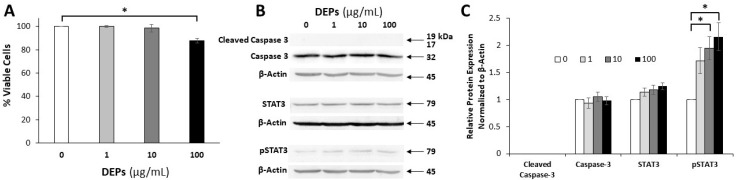
Cell viability assay (**A**) and Western blot analysis (**B**,**C**) for A549 cells after 24 h of incubation with DEPs at different concentrations (0, 1, 10, and 100 µg/mL). (**A**). The percentage of viable cells is reduced only after incubation with DEPs at 100 µg/mL. The difference was statistically significant (* *p* < 0.05). B. Protein expression analysis following incubation of A549 cells with DEPs at 0, 1, 10, and 100 µg/mL for 24 h. Proteins were extracted and 40 µg from each sample were processed for SDS-PAGE and Western blot using antibodies specific for caspase 3, the cleaved form of caspase 3, STAT3, and phosphorylated STAT3. Beta actin was used as the loading control. Note the pronounced up-regulation of the phosphorylated form of STAT3. **C.** Densitometric analysis of the protein bands is presented in the graph and shows significant up-regulation of the pSTAT3 (* *p* < 0.05).

Then the viability of A549 cells was tested using the CellTiter-Glo^®^ luminescent assay which showed some reduction in the percentage of viable cells after 24 h of incubation only at 100 µg/mL DEPs concentration. Statistical analysis showed that this decrease was significant (*p* < 0.05) when compared with control cells (Figure 9A).

To verify whether the reduction in the number of A549 cells when incubated with DEPs at 100 µg/mL is due to apoptosis, DEPs-treated cells were processed for protein extraction and Western blot analysis using antibodies specific for pro-caspase-3 and its cleaved form. There were no changes detected in the levels of caspase-3 and its cleaved form was not detected (Figure 9B). 

To test whether the phenotype of A549 cells is affected following incubation with DEPs at 1, 10, and 100 µg/mL for 24 h, we processed protein samples to detect any change in the expression patterns of STAT3 (Figure 9B). A gradual increase in the band density of the phosphorylated STAT3 was observed with increased concentration of DEPs from 1 to 10 µg/mL. At 100 µg/mL DEPs, the level of pSTAT3 remained high (Figure 9C).

## 4. Discussion

**Figure 10:**
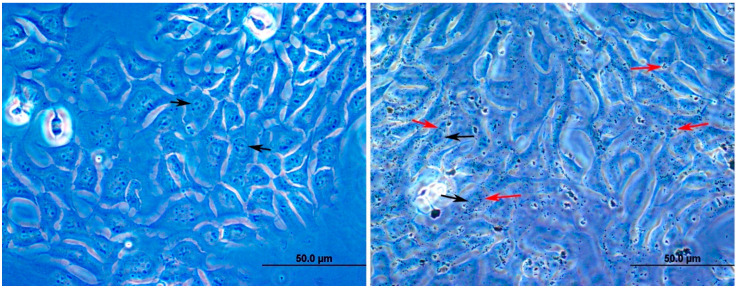
High magnification micrographs of mGS cells attached to glass coverslips following their incubation with 0 (**left**) and 10 (**right**) µg/mL DEPs for 24 h. The black arrows point to nuclei of mGS cells with prominent nucleoli. The two red arrows on the left side of the right images are pointing to the location of several fine DEPs that appear, with zooming in, to be at different focal planes. This might suggest that at least some of these DEPs are inside the cytoplasm of the cells. The two other red arrows on the right side of the image are pointing to small aggregates of DEPs surrounded by pale halos suggesting cytoplasmic endocytic bodies. Such structures are scattered in many cells. Magnification bar = 50 µm.

Little is known about the effects of DEPs on stem/progenitor cells. Exposure of stem/progenitor liver cells and lung cells to polycyclic aromatic hydrocarbons present in DEPs induce epigenetic toxicity characterized by a reduction in gap junctional intercellular communications and activation of MAPK activity and signaling pathways of both inflammation and carcinogenesis [31,32,33,34]. However, exposure of endothelial progenitor cells to DEPs impairs their number and migration and causes the formation of reactive oxygen species [35]. Consequently, inhibition of angiogenesis and a marked increase in atherosclerotic lesions occur. The current study demonstrates, in an in vitro model, the effects of the environmental DEPs on the immortal mGS cells with some emphasis on cell viability, migration, oxidative stress, and gene expression. Incubation of mGS cells with DEPs at 100 µg/mL caused a reduction in their number. This might indicate that DEPs inhibit the proliferation of mGS cells and/or induce their damage/death. However, only a few floating cells were occasionally observed in the wells treated with different concentrations of DEPs at any time point. Resistance to damage by DEPs was also observed in lung cells [36]. When lung epithelial cells were incubated with particulate matter for 72 h, electron microcopy revealed that diesel particles reacted with the cellular membranes and then internalized into the cytoplasm. However, the cells appeared healthy without any apoptotic changes. The main effects were increase of multilamellar bodies, formation of vacuoles containing particles, and development of atypical nuclei [36]. Even though electron microscopic examination were not carried out in the present study, nuclear atypia was not observed with DEPs incubation. Some mGS cells grown on coverslips were incubated with 0 (control) or 10 µg/mL DEPs for 24 h and then washed with PBS and fixed with 4% formaldehyde. Microscopic examination with 100× objective lens localized DEPs associated with the cells (Figure 10). 

In the current study, we incubated mGS cells with a wide range of concentrations of 1, 10, or 100 µg/mL DEPs for different time points. This dose of DEPs was established before in previous studies on erythrocytes [21] and also for another type of stem/progenitor cell that give rise to endothelial cells and was estimated based on the range of environmental daily concentration of DEPs [35]. Further work is needed to establish a dose of DEPs relevant to what human gastric cells are exposed to. Using this 1–100 µg dose, we have not detected any activation or upregulation in caspase 3, PARP and BCL2. These findings correlate with the negative effect of DEPs on oxidative stress. 

Three different oxidative stress markers are assessed in the present study: (i) GSH is the most abundant molecule among endogenous antioxidants, (ii) malondialdehyde is formed via peroxidation of polyunsaturated fatty acids and typically quantified using the TBARS assay, and (iii) NO regulates several biological functions including cellular differentiation and viability. In the current study, only a moderate increase in the level of NO was observed, but measurements of the levels of GSH and TBARS revealed no significant change when mGS cells are incubated with DEPs at 1, 10, or even 100 µg/mL for 24 h. This finding also indicates that mGS cells have some resistance to damage by these environmental particles. Indeed, there are evidences in the literature in support of stem cell resistance to toxins and these were attributed to their expression of drug transporter genes such as the ABCG cassettes, high capacity for DNA repair, and antioxidant effects [37,38,39]

Other body cells and tissues might be more vulnerable to environmental particles/toxins than mGS cells. Previous studies showed that exposure of mice to the hazard of smoking was associated with elevation in the levels of TBARS and a decrease of GSH concentrations, suggesting depletion of the antioxidants [40]. Using proteomic analysis in macrophages, a hierarchical oxidative stress in response to exposure to DEPs was demonstrated including a reduction in the level of GSH [41]. In a dopaminergic cell model, ultrafine DEPs induced a significant increase in the cellular nitrate level and the generation of reactive oxygen species leading to cytoplasmic dopamine accumulation [42]. Therefore, the oxidative stress plays a role in DEPs toxicity. The current study shows that this is not the case for mGS cells exposed to DEPs, probably because of their few small mitochondria and, therefore, little oxidative phosphorylation and free radical formation [11,14,43].

The quantitative real-time PCR was used to analyze changes in the expression pattern of some genes specific for the dynamics of mGS cells after exposure to DEPs. The focus was on genes responsible for the detoxification, proliferation, and death of stem cells. Glutathione S-transferases (GST) represent a family of enzymes that play an important role in detoxification of noxious agents by catalyzing their conjugation with reduced glutathione [44]. In the present study, incubating mGS cells with DEPs slightly upregulates the expression of GSTP1 gene to a level which is not enough to demonstrate an increase the levels of GSH released in the culture media. We also did not detect any significant change in the expression levels of the cell cycle-specific MKI67 gene of interphase and mitotic cells [45] after incubation of mGS cells with different concentrations of DEPs for 24 h.

Notch proteins represent a family of transmembrane proteins which are involved in direct cell-cell communication, thereby controlling cell differentiation [46,47]. Notch 3 expression was previously demonstrated in mGS cells [12]. In the present study, Notch 2 was also found to be expressed in mGS cells. Upon their exposure to DEPs, there was a decrease in the levels of Notch 2 and 3 proteins which might indicate alteration in the differentiation potential of mGS cells. In addition, BMI-1 proteins were down-regulated in mGS cells incubated with DEPs which might reflect changes in the self-renewal and/or pluripotency (stemness) of mGS cells. Interestingly down-regulations of Notch 2 and 3 are recently reported in the gastric epithelium of a transgenic mouse model with metaplastic changes [47]. These findings confirm that Notch proteins are involved in normal homeostasis of gastric stem cells and suggest that their down-regulation is associated with transformation into cancer stem cells with preneoplastic and eventual neoplastic transformations. In the current study, down-regulation of Notch proteins in mGS cells might also indicate their transformation into cancer stem cells and their potential to initiate cancer. This is in support of the hypothesis of the stem cell origin of cancer [48]. Indeed, mGS cells are tumorigenic after 3–4 months of their injection subcutaneously into nude mice, but they are neither carcinogenic nor metastatic [14]. 

STAT3 is a signal transducer, activator of transcription and a family member of latent cytoplasmic proteins [49]. This protein has been implicated in stem cell fate. It controls basal respiratory stem cells and regeneration of ciliated cells and also required to maintain the full differentiation potential of mammary gland stem cells [50,51]. In the present study, DEPs up-regulate and activate STAT3 in mGS cells in a dose dependent manner. A similar activation of STAT3 expression was previously reported when airway epithelial cells were incubated with DEPs [52]. Therefore, in the present study, exposure of mGS cells to DEPs affects various genes involved in the stemness of mouse gastric stem cells.

The mechanisms involved in the alteration of the stemness of mGS cells by incubation with DEPs require further investigations. One potential mechanism underlying this connection between DEPs exposure and the stemness of adult stem cells and subsequent increase in the incidence of cancer is DNA methylation, an epigenetic process with the capacity to integrate interactions between the environment and genes [31,53].

## 5. Conclusions

While it is well established that environmental DEPs have many toxic effects on cells and tissues of the cardio-pulmonary system [33,54], this study suggests that the stomach stem cells are also a target for DEPs. In an in vitro model of mGS cell exposure to DEPs, viability, migration, release of oxidative stress markers, and expression of some genes and proteins were systematically analyzed. While significant effects on cell viability only occurs at high concentration (100 µg/mL) of DEPs, the wound healing assay did not reveal any significant effects on the migration of mGS cells. This was associated with little or no change in the levels of oxidative stress markers. Expressions of proteins and mRNAs related to cell proliferation and apoptosis of stem cells were analyzed and revealed no significant changes, but there was alteration in some stem cell proteins. While Notch 2 and 3, and also Bmi-1, were down-regulated, STAT3 was activated. Therefore, the overall data suggest that DEPs have no or minor effects on the viability of gastric stem cells, but mostly target their stemness.
